# Chronic cough diagnosis, treatment, and referral practices among family physicians in the United States: a survey study

**DOI:** 10.1186/s12875-024-02433-1

**Published:** 2024-05-23

**Authors:** Joel B. Shields, Elisabeth Callen, Natalia Y. Loskutova, Jonathan Schelfhout, Christina M. Hester

**Affiliations:** 1 American Academy of Family Physicians National Research Network, 11400 Tomahawk Creek Parkway, Leawood, KS 66211 United States of America; 2 DARTNet Institute, 12635 East Montview Boulevard, Suite 129, Aurora, CO 80045 United States of America; 3 Merck, Sharpe, and Dohme Corp, 2000 Galloping Hill Road, Kenilworth, NJ 07033 United States of America

**Keywords:** Chronic cough, Persistent cough, Refractory chronic cough, Unexplained chronic cough, Primary care, Family medicine, Respiratory, Diagnosis

## Abstract

**Background:**

Cough is one of the most common presenting problems for patients in primary care and is largely managed in primary care clinical settings. Family physicians’ familiarity with chronic cough guidelines and the extent to which these guidelines translate into everyday practice have not been well described. The objective of this study was to characterize current diagnosis, treatment, and referral practices among family physicians and to identify potentially impactful strategies to optimize chronic cough management in primary care.

**Methods:**

We conducted a cross-sectional survey of 5,000 family physicians to explore diagnosis, treatment, and referral practices related to chronic cough management in adults in primary care. Respondents completed the survey via paper or online. The outcome measures were self-reported numeric ratings and responses related to the survey elements.

**Results:**

588 surveys were completed (11.8% response rate). About half (49.6%) of respondents defined chronic cough in a manner consistent with the American College of Chest Physicians (ACCP) chronic cough guidelines, with the rest differing in opinion primarily regarding duration of symptom presentation. Respondents reported trying to rule out most common causes of chronic cough themselves before referring (mean 3.41 on a 4-point scale where 4 is “describes me completely”) and indicated a desire for more resources to help them manage and treat chronic cough. Years in practice and rural/urban setting influenced diagnosis and referral practices.

**Conclusions:**

Family physicians see chronic cough as a complicated condition that can be and is often diagnosed and treated entirely in a primary care setting. They also value the ability to refer in complex cases. Our results support that family physicians provide evidence-based management of chronic cough.

**Supplementary Information:**

The online version contains supplementary material available at 10.1186/s12875-024-02433-1.

## Background

Cough is one of the most common presenting symptoms for patients in primary care and is a driver of referrals to secondary specialist care in the US and worldwide [1–4]. Chronic cough is more common in Europe, America, and Australia than in Asia than in other countries [5]. Cough lasting longer than eight weeks is considered chronic [1–3, 6–12] and has a prevalence of about 10% among adults in the United States [4, 9, 13, 14]. In a 2018 survey of US adults with chronic cough, 92% of respondents had visited a healthcare clinician in the prior six months, with the most (72%) visiting general and family practitioners [15].

Chronic cough can be a symptom of various diseases, and some physicians argue that, in some cases, it can be considered a respiratory condition in and of itself [9, 16]. The American College of Chest Physicians (ACCP) recommends a diagnosis algorithm based on patient history and examination and categorizes cough by duration: <3 weeks is considered acute; 3–8 weeks is subacute; and > 8 weeks is considered chronic [1, 7, 10–12]. The ACCP guidelines have been adopted by several different specialties and have been endorsed by the American Academy of Family Physicians (AAFP) for use in family medicine and primary care [12].

Importantly, chronic cough can result in problems beyond respiratory symptoms, creating a wide variety of challenges for the patient seeking treatment. Disturbed sleep, fatigue, and urinary incontinence – particularly among female patients – can impact the mental health and overall quality of life of those with chronic cough. Severe cases may lead to vomiting, muscle pain, rib fractures, and syncope, as well as embarrassment and social discomfort, particularly since the onset of the COVID-19 pandemic, making the patient feel stigmatized [7, 12, 17, 18].

Considering the prevalence of chronic cough and its extensive presentation and management in primary care, evidence-based assessment and treatment strategies must align with the needs of front-line primary care clinicians and their patients. Although much of the literature around chronic cough management is focused on other specialty settings, it is important to understand the current practices and needs of primary care clinicians regarding their important role in the front lines of effectively diagnosing and managing chronic cough. Here, we explored chronic cough diagnosis, treatment, and referral practices reported by family physicians to inform how best to support primary care clinicians and their patients in chronic cough management. We also examined how physician characteristics and practice context may influence management of chronic cough and self-reported educational needs.

## Methods

### Study design

This project was a cross-sectional survey of family physicians that was administered by the American Academy of Family Physicians (AAFP) National Research Network (NRN) from December 19, 2020, through March 19, 2021. The survey was sent to a random sample of 5,000 US-based family physician members of AAFP who were currently engaged in clinical practice for at least 50% of their professional time and cared for adult patients. Family physicians residing in Vermont were excluded due to state regulations. Respondents were compensated $10 for their time.

The survey was administered online using Qualtrics (Provo, Utah), with paper surveys mailed to those who did not respond to the initial emailed survey invitation. Personalized links were emailed through the AAFP blast email system. Two reminders were sent to non-responders at two-week intervals, and a paper survey was mailed to all non-responders two weeks after the first email invitation. Data collection concluded three months following initial contact.

### Survey instrument

The survey was developed for this study in consultation with a panel of subject matter experts consisting of six family physicians, three allergy/immunologists, and one pulmonologist [see Additional File 1 for survey instrument]. These experts identified key variables of interest and relevance to management of chronic cough, such as defining, diagnosing, and treating chronic cough; the when and how of referring chronic cough patients to specialists; and educational needs related to chronic cough. All experts contributed to development and review of survey items. The final draft survey was pilot tested by the family physicians on the expert panel for clarity and ease of completion and was refined further with their feedback. The survey included questions on demographic characteristics of both the respondent and their practice as well as questions with Likert-type response options regarding how they characterize chronic cough prior to being provided a definition; the usefulness of various indicators and diagnostics when evaluating and diagnosing chronic cough; and how they would rank several problems that patients with chronic cough report as reasons for seeking care, Numerical values were assigned to Likert-type response options to create ordinal data for each question, allowing for calculation of means and standard deviations for responses for each question. See table footnotes for each specific variable for responses associated with numerical values.

### Data cleaning, statistics, and analytical methods

Completed paper surveys were scanned via a Fujitsu FI-6770 scanner and checked for accuracy. Data from paper and online surveys were merged into one combined dataset and checked to ensure data quality and completeness. Duplicates were removed, with paper survey responses used if both surveys were completed. Descriptive statistics were prepared for all questions. All available data was used, assuming ignorable missingness (missing completely at random, MCAR). For MCAR, pairwise deletion (i.e., all available data are used, leading to different samples for each question) was used when reporting on all included results. For the initial analyses, Student’s/Welch’s t-tests, ANOVAs (ANalysis of VAriance), and ANCOVAs (ANalysis of CoVAriance) were used for inferential statistics and focusing on the overall variables/values. Using the results of the initial analyses, we identified specific demographic variables for further analysis. For ANOVAs and ANCOVAs, post-hoc tests were completed for significant overall results to determine possible differences between the pairs of categories. Tukey’s HSD (Honestly Significant Difference) was used when there was homogeneity in the variance, and Games-Howell was used when homogeneity in the variance was violated. Statistical testing was two-sided with alpha = 0.05 and *p*-values reported. All statistical analyses were performed using SPSS 25 (Armonk, NY).

## Results

### Demographics

A total of 588 (11.8%) respondents completed surveys, with 410 (69.7%) of the respondents completing on paper and 178 (30.3%) online (Table 1). These numbers are in line with other survey studies we have completed [19–23]). The majority of the respondents were born in 1970 or later (54.2%), female (52.8%), not Hispanic or Latinx (91.8%), white (74.4%), out of residency for 16 or more years (51.5%), spent 76–99% of their clinical time with adults (54.8%), and worked in an urban setting (84.4%). The respondents worked for a variety of employers including private non-profit hospital or health system (32.0%), physician group (19.3%), or for themselves (18.1%). The respondents had similar characteristic distributions as the overall AAFP membership: average age and rurality were almost equal, our sample has slightly more females and skews older than the AAFP membership [24].

**Table 1:** Participant demographics

Characteristics	*n*, %
***Year of Birth (n = 539)***	
1945–1959	104, 19.3
1960–1969	143, 26.5
1970–1979	124, 23.0
1980–1993	168, 31.2
***Gender (n = 587)***	
Male	275, 46.8
Female	310, 52.8
Other	0, 0.0
Prefer not to answer	2, 0.3
***Ethnicity (n = 582)***	
Hispanic or Latinx	23, 4.0
Not Hispanic or Latinx	534, 91.8
Prefer not to answer	25, 4.3
***Race (n = 585)***	
American Indian or Alaskan Native	2, 0.3
Native Hawaiian or Other Pacific Islander	1, 0.2
Asian	85, 14.5
Black or African American	25, 4.3
White	435, 74.4
Multiracial	12, 2.1
Other	8, 1.4
Prefer not to answer	17, 2.9
***Years Since Residency (n = 584)***	
0	25, 4.3
1–5	111, 19.0
6–10	72, 12.3
11–15	75, 12.8
16–20	65, 11.1
More than 20	236, 40.4
***Primary Employer (n = 587)***	
Self*	106, 18.1
Physician group (single- or multi-specialty)	113, 19.3
University-owned (public or private) clinic or hospital	46, 7.8
Private for-profit hospital or health system	40, 6.8
Private non-profit hospital or health system	188, 32.0
Federal, state, or local government, community board, etc. (excluding universities)	47, 8.0
Other	47, 8.0
***Rurality (n = 442)***	
Urban	373, 84.4
Rural	69, 15.6

*Self-employed, majority practice owner, independent contractor, etc.

### Descriptive survey findings

#### Definition of chronic cough

Prior to being presented with a definition, most respondents indicated that a chronic cough should last 8 or more weeks (84.2%), be persistent on most days (66.0%), and have a noticeable effect on a patient’s daily life (54.0%) (Table 2). Phlegm/sputum were not considered relevant for defining chronic cough (98.5%). Respondents were then presented with the definition of chronic cough as a cough that lasts 8 weeks or longer, and the majority agreed or strongly agreed with this definition (77.2%; Table 2).

**Table 2:** How family physicians identify and define chronic cough

Response Category	*n*, %
**From the choices below, please select the ones that help you confirm a patient’s cough is chronic. I decide a cough is chronic based on its:**	
*Duration (choose one): (n = 560)*	
More than 2 weeks	23, 4.1
More than 3 weeks	65, 11.6
More than 8 weeks	278, 49.6
More than 3 months	194, 34.6
Other	16, 2.8
***Number of days per week a person coughs (choose one): (n = 564)***	
Persistent on most days, if not every day	372, 66.0
Happens more days than not	176, 31.2
Is not relevant	23, 4.1
***Phlegm/Sputum (choose one): (n = 524)***	
Must be present	8, 1.5
Is not relevant	516, 98.5
***Effect on patient’s daily life (choose one): (n = 537)***	
Must be noticeable	290, 54.0
Is not relevant	247, 46.0
***Other: (n = 588)***	
Other	79, 13.4
**To what extent do you agree or disagree with the following definition of chronic cough? Chronic cough is a cough that lasts eight weeks or longer in adults.**	
Strongly disagree	49, 8.6
Disagree	43, 7.5
Neither agree nor disagree	38, 6.6
Agree	308, 53.8
Strongly agree	134, 23.4

#### Chronic cough evaluation, diagnosis, and treatment

Respondents rated the following items useful for evaluation and diagnosis (in order of mean score from most to least useful (Table 3)): smoking status, medication history [especially angiotensin converting enzyme inhibitors (ACEs) and angiotensin receptor blockers (ARBs], exposure to environmental irritants, spirometry if asthma is suspected, and pulmonary function studies. For treatment, “trial of other medication” was considered useful, higher than the two medications listed on the survey: pregabalin and gabapentin (Table 3). The “other medication” category included medications sometimes used for treatment such as proton pump inhibitors, inhaled corticosteroids, inhalers, antihistamine, allergy meds, cough suppressant, and gastroesophageal reflux disorder medications (not shown).

**Table 3:** Ranking usefulness of strategies family physicians use for chronic cough, evaluation, diagnosis, and treatment

	*n*	Mean (SD)	Not at all useful	Slightly useful	Moder-ately useful	Very useful	Extreme-ly useful	Do not use	
***Medical History***:									
Smoking status	561	4.80 (0.44)	0,	0,	9,	96,	452,	4,	
			0	0	1.6	17.1	80.6	0.7	
Medication history (ACE/ARBs)	561	4.62 (0.61)	0,	6,	20,	154,	377,	4,	
			0	1.1	3.6	27.5	67.2	0.7	
Exposure to environmental irritants	562	4.52 (0.68)	1,	5,	38,	173,	343,	2,	
			0.2	0.9	6.8	30.8	61	0.4	
Other	130	3.96 (1.11)	4,	4,	21,	24,	37,	40,	
			3.1	3.1	16.2	18.5	28.5	30.8	
Lifestyle	562	3.95 (0.89)	2,	31,	129,	229,	170,	1,	
			0.4	5.5	23	40.7	30.2	0.2	
Profession/Occupation	561	3.85 (0.93)	4,	43,	135,	226,	151,	2,	
			0.7	7.7	24.1	40.3	26.9	0.4	
***Diagnostics/Testing***:									
Spirometry, if asthma is suspected	560	4.01 (0.91)	1,	33,	121,	206,	195,	4,	
			0.2	5.9	21.6	36.8	34.8	0.7	
Pulmonary function studies	560	3.99 (0.82)	0,	21,	126,	244,	165,	4,	
			0	3.8	22.5	43.6	29.5	0.7	
Physical examination	561	3.92 (0.94)	2,	34,	155,	185,	183,	2	
			0.4	6.1	27.6	33	32.6	0.4	
Chest radiography/Chest x-ray	559	3.77 (0.98)	2,	59,	155,	191,	149,	3,	
			0.4	10.6	27.7	34.2	26.7	0.5	
Allergy assessments	558	3.46 (0.95)	7,	76,	203,	187,	80,	5,	
			1.3	13.6	36.4	33.5	14.3	0.9	
Chest CT scans	557	3.13 (1.06)	22,	145,	195,	122,	68,	5,	
			3.9	26	35	21.9	12.2	0.9	
Cardiac echo	552	2.71 (0.95)	42,	191,	205,	74,	24,	16,	
			7.6	34.6	37.1	13.4	4.3	2.9	
Sinus CT scans	558	2.54 (0.97)	68,	208,	186,	56,	20,	20,	
			12.2	37.3	33.3	10	3.6	3.6	
Blood tests	557	2.29 (0.95)	91,	279,	117,	38,	19,	13,	
			16.3	50.1	21	6.8	3.4	2.3	
***Referral/Treatment/Management***:									
Referral to a specialist	559	3.58 (0.93)	2,	66,	200,	186,	102,	3,	
			0.4	11.8	35.8	33.3	18.2	0.5	
Trial of other medication	420	3.40 (1.08)	15,	58,	134,	97,	71,	45,	
			3.6	13.8	31.9	23.1	16.9	10.7	
Speech therapy	540	2.47 (0.99)	65,	192,	120,	61,	12,	90,	
			12	35.6	22.2	11.3	2.2	16.7	
Trial of pregabalin	550	1.87 (0.90)	144,	140, 25.5	53,	18,	3,	192,	
			26.2		9.6	3.3	0.5	34.9	
Trial of gabapentin	548	1.75 (0.95)	138,	148,	58,	24,	5,	175, 31.9	
			25.2	27	10.6	4.4	0.9		

Note: 1 = Not at all useful; 2 = Slightly useful; 3 = Moderately useful; 4 = Very useful; 5 = Extremely useful

Respondents indicated slight disagreement with statements stating they have a good understanding of the causes of chronic cough and that they are confident in their abilities to evaluate and treat chronic cough (Table 4). In addition, respondents indicated they were interested in more resources and education on evidence-based management strategies, treatment options, recommended diagnostics as well as patient resources (education, shared decision-making support, and information on patient-centered care; Table 4).

**Table 4:** Referral practices, educational needs, and patient considerations

	Question & Response Options	*n*	Mean (SD)
**Assessment and Referral**	**To what extent do the approaches listed below describe or not describe the way you assess and treat chronic cough (in descending order)?** **Scale: 1 = Does not describe me at all; 2 = Describes me slightly; 3 = Describes me mostly; 4 = Describes me completely**		
	I try to assess for and to rule out most common causes of chronic cough through tests and trials of medications before I refer to a specialist.	554	3.41 (0.61)
	I conduct my evaluation in phases, starting with medical history and few diagnostic tests and using several trials of medications before I order more expensive tests or refer to a specialist.	555	3.28 (0.61)
	I try to assess and treat the upper airway cough syndrome first and consider more expensive and invasive tests for those who are not improving.	553	3.08 (0.71)
	I follow evidence-based guidelines offered by the American College of Chest Physicians (ACCP) for management of chronic cough.	535	2.46 (0.87)
	I refer all or most patients with chronic cough to a specialist for full evaluation.	548	1.52 (0.65)
	**If you refer your patients with chronic cough, how often do you refer your patients to the following specialists or services (in descending order)?** **Scale: 1 = Never; 2 = Rarely; 3 = Sometimes; 4 = Often; 5 = Always**		
	Pulmonologist	557	3.43 (0.69)
	Allergist	549	3.08 (0.76)
	Otorhinolaryngology	545	2.89 (0.83)
	Gastroenterologist	550	2.66 (0.77)
	Speech therapy	539	1.84 (0.87)
	Oncologist	537	1.77 (0.66)
	Internal Medicine / Infectious Disease	530	1.58 (0.65)
	Behavioral therapy	537	1.57 (0.75)
	Other	55	1.49 (0.98)
**Educational Needs**	**Please select how much you agree or disagree with each statement below (in descending order).** **Scale: 1 = Strongly disagree; 2 = Disagree; 3 = Neither agree nor disagree; 4 = Agree; 5 = Strongly agree**		
	I have a good understanding of the mechanisms and underlying conditions causing chronic cough	571	2.89 (0.96)
	I feel confident in my ability to evaluate and treat chronic cough	569	2.84 (1.02)
	**To what extent would education in the following areas be beneficial to you (in descending order)?** **Scale: 1 = Not at all beneficial; 2 = Slightly beneficial; 3 = Beneficial; 4 = Mostly beneficial; 5 = Very beneficial**		
	Evidence-based practices for management of chronic cough	554	4.15 (0.91)
	Treatment options for chronic cough	552	4.07 (0.90)
	Recommended diagnostic tests	550	3.82 (0.91)
	Patient education and communication strategies	553	3.69 (1.02)
	Shared decision making	554	3.52 (1.09)
	Effects of chronic cough on patient’s well-being	552	3.27 (1.09)
**Patient Considerations**	**Please rank in the order of prevalence the following problems for why patients with chronic cough seek care in your clinic? (Note: Higher number means lower rank)**		
	Patient cannot tolerate it anymore	542	2.03 (1.23)
	Patient concerns that it is cancer or something serious	541	2.44 (1.40)
	Patient’s family cannot tolerate it anymore	531	3.59 (1.49)
	Negative effects on physical health (e.g., headaches, poor sleep, vomiting, wetting/soiling pants, pain)	531	3.77 (1.54)
	Negative effects on work or school	528	4.43 (1.30)
	Stigma and self-consciousness	524	4.76 (1.51)
	Other	165	6.61 (1.30)
	**How frequently do your patients report that they have experienced the following due to their chronic cough (in descending order)?** **Scale: 1 = Never; 2 = Rarely; 3 = Half of the time; 4 = Often; 5 = Very often**		
	Stigma that any cough is COVID-19	558	3.35 (1.07)
	Sleep disturbances	558	3.24 (0.88)
	Social embarrassment or being uncomfortable in public	560	3.16 (0.98)
	Anxiety	558	2.72 (0.94)
	Loss of bladder control (urinary incontinence)	558	2.70 (0.88)
	Chest pain	558	2.59 (0.84)
	Difficulties with speaking on the phone or in-person	557	2.57 (0.93)
	Headaches	557	2.52 (0.85)
	Depression	551	2.19 (0.82)
	Vomiting	557	2.07 (0.65)
	Fractured ribs	557	1.58 (0.54)

#### Referral to specialty care

Family physicians indicated at least sometimes referring chronic cough patients to specialists. In order of most to least, patients were referred to pulmonologists, allergists, otorhinolaryngologists, and gastroenterologists (Table 4). Referrals to other specialties were less frequent. Most respondents indicated conducting their evaluation in phases and assessing for and ruling out most common causes of chronic cough before referral to a specialist. They reported assessing and treating for upper airway cough syndrome first, and their responses indicated they follow ACCP guidelines. Notably, respondents indicated little identification with the statement, “I refer all or most patients with chronic cough to a specialist for full evaluation” (Table 4).

#### Perceptions of patient experience

Respondents indicated “patient cannot tolerate it anymore” and “patient concern that it is cancer or something serious” as the first and second most common reason patients with chronic cough sought care, respectively (Table 4). Respondents also indicated their perceptions of how frequently patients with chronic cough report physical and social challenges related to their cough symptoms, such as incontinence or stigma that cough may be due to COVID-19 (Table 4).

### Results of statistical analyses

Two demographic characteristics emerged as variables related to differences in responses: rurality (rural or urban) and years since residency (0, 1–5, 6–10, 11–15, 16–20, or more than 20 years). Only significant results for each category are presented below (Table 5).

**Table 5:** Statistical results

***T-Tests***		
	Mean (SD)	statistics (sig)
To what extent do you agree or disagree with the following definition of chronic cough? Chronic cough is a cough that lasts eight weeks or longer in adults. (*N* = 433)		
Urban Respondents	3.63 (1.20)	t: -2.236 (0.027)
Rural Respondents	3.94 (1.03)	
Please select how much you agree or disagree with each statement below: I feel confident in my ability to evaluate and treat chronic cough. (*N* = 429)		
Urban Respondents	2.85 (1.05)	t: -2.263 (0.026)
Rural Respondents	2.58 (0.84)	
To what extent do the approaches listed below describe or not describe the way you assess and treat chronic cough? I refer all or most patients with chronic cough to a specialist for full evaluation. (*N* = 430)		
Urban Respondents	1.54 (0.66)	t: 2.539 (0.013)
Rural Respondents	1.34 (0.54)	
***ANOVAs***		
How useful are the following in evaluating and diagnosing chronic cough? Diagnostics/Testing: Chest radiography/Chest x-ray. (*N* = 550)		
0 years	3.40 (1.08)	F: 2.681 (0.021)
1–5 years	3.61 (0.95)	
6–10 years	3.80 (0.96)	
11–15 years	3.95 (0.95)	
16–20 years	3.63 (0.98)	
More than 20 years	3.89 (0.99)	
How useful are the following in evaluating and diagnosing chronic cough? Diagnostics/Testing: Allergy Assessments (*N* = 549)		
0 years	3.36 (1.00)	F: 2.322 (0.042)
1–5 years	3.28 (0.92)	
6–10 years	3.39 (0.98)	
11–15 years	3.74 (0.92)	
16–20 years	3.49 (0.99)	
More than 20 years	3.54 (0.98)	
*Tukey Post Hoc Tests: Significant difference (p < 0.05) between 1–5 years and 11–15 years.*		
If you refer your patients with chronic cough, how often do you refer you patients to the following specialists or services? Behavioral Therapy (*N* = 536)		
0 years	1.62 (0.77)	F: 2.289 (0.049)
1–5 years	1.62 (0.76)	
6–10 years	1.51 (0.70)	
11–15 years	1.35 (0.61)	
16–20 years	1.54 (0.63)	
More than 20 years	1.63 (0.82)	
Tukey Post Hoc Tests: Significant difference (*p* < 0.05) between 11–15 years and more than 20 years.		
***ANCOVAs***		
To what extent do the approaches listed below describe or not describe the way you assess and treat chronic cough: I refer all or most patients with chronic cough to a specialist for full evaluation. *Controlled for: Years Since Residency* (*N* = 409)		
Urban Respondents	1.54 (0.66)	F: 5.172 (0.023)
Rural Respondents	1.34 (0.54)	
How useful are the following in evaluating and diagnosing chronic cough? Diagnostics/Testing: Chest radiography/Chest x-ray. *Controlled for: Rurality* (*N* = 417)		
0 years	3.20 (1.08)	F: 2.562 (0.027)
1–5 years	3.56 (0.92)	
6–10 years	3.78 (0.92)	
11–15 years	3.86 (0.94)	
16–20 years	3.61 (1.02)	
More than 20 years	3.89 (0.99)	

#### Definition

When provided with the ACCP definition that “chronic cough is a cough that lasts eight weeks or longer in adults,” the respondents located in urban areas agreed to a lesser extent with the definition than the respondents located in rural areas (Table 5).

#### Diagnosis and treatment

For diagnosis, two strategies differed significantly in uptake: chest radiography/chest x-ray and allergy assessments. There was a significant difference among groups of respondents with differing years since residency (no significant Tukey HSD Post Hoc Tests; Table 5) for the respondents who used chest radiography/chest x-ray as part of evaluating and diagnosing chronic cough, with increasing experience corresponding to increased uptake of chest radiography/chest x-ray. After controlling for respondents’ urban or rural location, respondents with more years since residency were still more likely to use chest radiography/chest x-ray than respondents with fewer years since residency (no significant Tukey HSD Post Hoc Tests; Table 5). There was also a significant difference in the use of allergy assessments among respondent groups with differing years since residency. Those who were 11–15 years since residency were more likely to include allergy assessments as part of their diagnostics and testing for chronic cough than those in 1–5 years post-residency (Table 5).

#### Referral

There was a significant difference in the use of Behavioral Therapy among respondent groups with differing years since residency (Table 5). For the Tukey HSD Post Hoc Tests, the group with more than 20 years since residency was more likely to refer to behavioral therapy than the group with 11–15 years since residency.

While the respondents located in urban settings were more likely than respondents located in rural settings to indicate they refer to a specialist for full evaluation (Table 5), respondents located in urban areas indicated they had more confidence than the respondents located in rural areas in evaluating and treating chronic cough. While controlling for respondents’ years since residency, respondents located in urban settings were still more likely than respondents located in rural settings to indicate they refer to a specialist for full evaluation (Table 5).

## Discussion

Treatment guidelines for family physicians take multiple factors into account when defining and diagnosing chronic cough, such as duration, frequency, impact on daily life, and results of clinical and empirical assessments [12]. Interestingly, while we found that half of the family physician respondents to our survey apply the ACCP definition of longer than 8 weeks in duration [1–3, 6–12], one-third reported using three months (Table 2). Further exploration into the use of a longer duration may shed light into the clinical insights used by family physicians in determining when to consider cough chronic. In addition to considering cough duration, family physician respondents take patient-centered factors into account such as number of days per week that a patient coughs and the impact of cough on daily life such as sleep disruption, urinary incontinence, and impact on family members. These considerations are consistent with literature describing the primary reasons patients seek medical care for chronic cough [7, 12, 17, 18].

Our findings support that chronic cough is largely managed within primary care without referral with respondents indicating a preference to treat most chronic cough patients themselves whenever possible, as is supported by other studies [4, 9]. Consistent with this finding and prior studies, we found that primary care physicians refer patients with chronic cough after exhausting resources at their disposal [12, 14]. It is important to note that survey respondents reported that many if not most chronic cough patients are being managed by family physicians in primary care and that, for those referred to other specialists, family physicians continue to manage the comprehensive care for these patients. Studies examining chronic cough may need to differentiate between those patients managed effectively in primary care and those requiring management in collaboration with other specialists.

Here, we show that family physicians are comfortable managing chronic cough because they reported a general understanding of the most common causes of and treatments for chronic cough, consistent with existing recommendations [8–10, 12]. Interestingly, despite comfort with managing and treating chronic cough themselves and adherence to appropriate guidelines, only a minority agreed or strongly agreed that they were confident in their abilities to evaluate and treat this condition. This apparent lack of confidence suggests a potential need for education to validate and confirm family physician knowledge about chronic cough management strategies. It may also help explain why we found that family physicians value the ability to refer as an important tool in chronic cough management.

Years of experience since residency related to differential approaches to both diagnosis and referral for chronic cough. These differences, specifically for chest x-rays, allergy assessments, and referrals to behavioral therapy (Table 5), may have been influenced by differing training experiences and evolving clinical guidance during both residency and years in practice [25, 26]. Understanding and addressing difference in practice that may result from timing of training could inform a framework for ensuring up-to-date continuing medical education is available to promote current best practices.

Referral to other specialists was influenced by rural/urban practice location. Rural physicians reported referring less often and indicated less satisfaction with the availability of specialists in their area. Interestingly, rural physicians reported having less confidence in evaluating and treating chronic cough than those in urban areas, possibly the result of having to manage more complex cases without referral (Table 5). This is consistent with the American Board of Family Medicine’s finding that overall, rural family physicians have a broader scope of practice than urban physicians, likely due to less overall infrastructure support which may include fewer referral options [27]. This study was not designed to focus on the matter of rural specialist availability, but our findings suggest the need for further exploration of whether/how rural physicians manage chronic cough differently than those in urban areas.

### Limitations

There are several limitations inherent with survey research that should be noted. Non-response bias can be an issue with any voluntary survey and can limit the generalizability of results, and risk for bias can be greater with lower response rates. While the sample of 5,000 is unlikely to create issues due to random error, the response rate of 11.8% carries with it a risk of non-random error. There may be differences between practices and perspectives of family physicians who completed the survey and those who chose not to complete it. In addition, self-report of behaviors comes with its own limitations, as can recall bias. We worked to develop a survey that is unambiguous and elicits reliable results, but we also cannot discount the possibility of a degree of bias from subjective perceptions. It should also be noted that this is a US-based survey, so the results may not be applicable to physicians outside the United States.

## Conclusions

Family physicians use multiple factors to determine when a cough is chronic, but when prompted, generally agree with the ACCP guidelines of longer than 8 weeks. Family physicians prefer to treat chronic cough themselves and report a patient-centered approach to caring for patients with chronic cough, including referral to specialists (particularly pulmonologists) when appropriate. Family physicians in rural contexts tended to report referring less often, possibly reflecting their adaptation to a need for a broader scope of practice in rural primary care. Overall, family physicians report providing evidenced-based management of chronic cough.

### Electronic supplementary material

Below is the link to the electronic supplementary material.

Supplementary Material 1

## Data Availability

The data that support the findings of this study are available from the authors. Restrictions apply to the availability of these data, which are available upon request but will not be made publicly available. Only deidentified datasets will be shared.

## Abbreviations

AAFP: American Academy of Family Physicians
ACCP: American College of Chest Physicians
ACE: Angiotensin Converting Enzyme
ANCOVA: ANalysis of CoVAriance
ANOVA: ANalysis of VAriance
ARB: Angiotensin Receptor Blocker
HSD: Honestly Significance Test
MCAR: Missing Completely at Random
NRN: National Research Network
