# Supplementary material for: Chronic cough diagnosis, treatment, and referral practices among family physicians in the United States: a survey study
Source: BMC Prim Care. 2024 May 23;25:181. doi: 10.1186/s12875-024-02433-1 (PMC11112819; doi:10.1186/s12875-024-02433-1)
Supplement: Supplementary file 1 — Supplementary Material 1 [file 12875_2024_2433_MOESM1_ESM.docx]

**Additional File 1: Survey Instrument**

**The American Academy of Family Physicians (AAFP) National Research Network is conducting a survey study designed to better understand how clinicians assess and manage patients with chronic cough within their clinic as well as understand clinicians' knowledge, confidence, and beliefs surrounding the treatment and management of chronic cough. 

Your participation is voluntary. By completing the survey, you consent to participate in this research. If you do not wish to answer any question, you may skip it and go to the next question. At any time, you have the option to withdraw. There is no penalty or loss of benefits for not participating or for discontinuing your participation.

If you have any questions about this survey please contact Joel Shields, Project Manager, at (913) 906-6152 or jshields@aafp.org. If you have any questions about your rights as a research participant, please contact Jennifer Farris, AAFP Institutional Review Board, at (913) 906-6134 or jfarris@aafp.org.

The survey will take approximately 15-20 minutes. Your responses will be kept confidential. There are no right or wrong answers. Thank you for taking this survey!**

**1. Please provide your year of birth.**

________________________________________________________________

**2. Please select your gender.**

- Male (1)
- Female (2)
- Other (3)
- Prefer not to answer (4)

**3. Please select your ethnicity.**

- Hispanic or Latinx (1)
- Not Hispanic or Latinx (2)
- Prefer not to answer (3)

**4. Please select your race. (Select one)**

- American Indian or Alaskan Native (1)
- Native Hawaiian or Other Pacific Islander (2)
- Asian (3)
- Black or African American (4)
- White (5)
- Multiracial (6)
- Other (please specify): (7) ________________________________________________
- Prefer not to answer (8)

**5. How many years has it been since you graduated from residency?**

- 0 (1)
- 1-5 (2)
- 6-10 (3)
- 11-15 (4)
- 16-20 (5)
- More than 20 (6)
- Not applicable (7)

**6. Who is your primary employer? (Select one)**

- You (self-employed, majority practice owner, independent contractor, etc.) (1)
- Physician group (single- or multi-specialty) (2)
- University-owned (public or private) clinic or hospital (3)
- Private for-profit hospital or health system (4)
- Private non-profit hospital or health system (5)
- Managed care organization or insurance company (6)
- Federal, state, or local government, community board, etc. (not including universities) (7)
- Locum tenens group/staffing organization (8)
- Medical school (9)
- Other (please specify): (10) _______________________________________________

**7. What is the state or territory where you practice?
Note: This information is required for data processing.**

_____________________________

If you practice in Vermont, we cannot send you a gift card. You may either complete the survey without a reward or stop now and return the survey.

**8. How much of your time in practice is spent caring for patients that are 18 years or older?**

- 0% of the time (1)
- 1% to 25% of the time (2)
- 26% to 50% of the time (3)
- 51% to 75% of the time (4)
- 76% to 99% of the time (5)
- 100% of the time (6)

*If you do not see patients 18 years or older, do not proceed any further and return the survey.*

**9. How many adult patients do you see in a typical week (best estimate is acceptable)?**

________________________________________________________________

**10. From the choices below, please select the ones that help you confirm a patient’s cough is chronic.**

**I decide the cough is chronic based on its:**

**Duration (choose one):**

- More than 2 weeks (1)
- More than 3 weeks (2)
- More than 8 weeks (3)
- More than 3 months (4)
- Other (please specify): (5) ________________________________________________

**Number of days per week a person coughs (choose one):**

- Persistent on most days, if not every day (6)
- Happens more days than not (7)
- Is not relevant (8)

**Phlegm/Sputum (choose one):**

- Must be present (9)
- **Is not relevant (10)**

**Effect on patient’s daily life (choose one):**

- Must be noticeable (11)
- Is not relevant (12)

**Other:**

- Other (please specify): (13)

**11. What word do you most frequently use to describe a chronic cough that persists despite assessment and treatment according to current guidelines? (Select one)**

- Unexplained (1)
- Refractory (2)
- Idiopathic (3)
- Persistent (4)
- Psychogenic (5)
- Habit (6)
- Tic (7)
- Other (please specify): (8) ________________________________________________

**Note: For the rest of the questions in this survey please consider only your adult (18 years or older) patients and use the definition of chronic cough as *a cough that lasts eight weeks or longer*.**

**12. To what extent do you agree or disagree with the following definition of chronic cough?**
**Chronic cough is a cough that lasts eight weeks or longer in adults.**

- Strongly disagree (1)
- Disagree (2)
- Neither agree nor disagree (3)
- Agree (4)
- Strongly agree (5)

**13. How many adult patients with pulmonary disease who have chronic cough do you see in a typical week (best estimate is acceptable)?**

- 0 (1)
- 1-3 (2)
- 4-6 (3)
- 7-9 (4)
- 10 or more (5)

**14. What is the percentage of patients with unexplained (or refractory) chronic cough in your practice (best estimate)?**

**15. Please select how much you agree or disagree with each statement below.**

|  | Strongly disagree (1) | Disagree (2) | Neither agree nor disagree (3) | Agree (4) | Strongly Agree (5) |
| --- | --- | --- | --- | --- | --- |
| Chronic cough is a distinct disease | ○ | ○ | ○ | ○ | ○ |
| Chronic cough is a symptom of an underlying disease | ○ | ○ | ○ | ○ | ○ |
| Patients with chronic cough usually get better over time without treatment | ○ | ○ | ○ | ○ | ○ |
| Patients with chronic cough usually get worse over time without treatment | ○ | ○ | ○ | ○ | ○ |
| There is not much that can be done for patients' complaints about chronic cough | ○ | ○ | ○ | ○ | ○ |
| Most unexplained cough in adults is psychogenic and should be evaluated by mental/behavioral health | ○ | ○ | ○ | ○ | ○ |

**16. Please select how much you agree or disagree with each statement below.**

|  |  |  |  |  |  |
| --- | --- | --- | --- | --- | --- |
|  | Strongly disagree (1) | Disagree (2) | Neither agree nor disagree (3) | Agree (4) | Strongly agree (5) |
| I have a good understanding of the mechanisms and underlying conditions causing chronic cough | ○ | ○ | ○ | ○ | ○ |
| Family Medicine should be primarily responsible for management of patients with chronic cough | ○ | ○ | ○ | ○ | ○ |
| I feel confident in my ability to evaluate and treat chronic cough | ○ | ○ | ○ | ○ | ○ |
| Chronic cough is a frequent problem in primary care | ○ | ○ | ○ | ○ | ○ |

**17. Please rank in the order of prevalence the following problems for why patients with chronic cough seek care in your clinic?**

______ Patient cannot tolerate it anymore (1)

______ Patient's family cannot tolerate it anymore (2)

______ Patient concerns that it is cancer or something serious (3)

______ Negative effects on work or school (4)

______ Negative effects on physical health (e.g., headaches, poor sleep, vomiting, wetting/soiling pants, pain) (5)

______ Stigma and self-consciousness (6)

______ Other (please explain): (7)

**18. How frequently do your patients report that they have experienced the following due to their chronic cough?**

|  | Never (1) | Rarely (2) | Half of the time (3) | Often (4) | Very Often (5) |
| --- | --- | --- | --- | --- | --- |
| Loss of bladder control (urinary incontinence) | ○ | ○ | ○ | ○ | ○ |
| Chest pain | ○ | ○ | ○ | ○ | ○ |
| Sleep disturbances | ○ | ○ | ○ | ○ | ○ |
| Headaches | ○ | ○ | ○ | ○ | ○ |
| Vomiting | ○ | ○ | ○ | ○ | ○ |
| Fractured ribs | ○ | ○ | ○ | ○ | ○ |
| Anxiety | ○ | ○ | ○ | ○ | ○ |
| Depression | ○ | ○ | ○ | ○ | ○ |
| Stigma that any cough is COVID-19 | ○ | ○ | ○ | ○ | ○ |
| Social embarrassment or being uncomfortable in public | ○ | ○ | ○ | ○ | ○ |
| Difficulties with speaking on the phone or in-person | ○ | ○ | ○ | ○ | ○ |

**19. How would you rate the following areas for the majority of your patients with chronic cough?**

|  | Poor (1) | Fair (2) | Average (3) | Good (4) | Excellent (5) |
| --- | --- | --- | --- | --- | --- |
| Adherence to recommended medication treatments | ○ | ○ | ○ | ○ | ○ |
| Adherence to recommended physical and psychological interventions such as speech therapy, behavioral suppression, respiratory retraining and other self-management techniques | ○ | ○ | ○ | ○ | ○ |
| Engagement in shared decision making | ○ | ○ | ○ | ○ | ○ |
| Adherence to recommended follow-up visits | ○ | ○ | ○ | ○ | ○ |
| Access to and availability of necessary treatments | ○ | ○ | ○ | ○ | ○ |
| Access to and availability of specialists (e.g., pulmonary, allergy, otolaryngologist) in your area | ○ | ○ | ○ | ○ | ○ |

**20. How useful are the following in evaluating and diagnosing chronic cough?**

|  | Not at all useful (1) | Slightly useful (2) | Moderately useful (3) | Very useful (4) | Extremely useful (5) | Do not use (6) |
| --- | --- | --- | --- | --- | --- | --- |
| **Medical History:** |  |  |  |  |  |  |
| Lifestyle | ○ | ○ | ○ | ○ | ○ | ○ |
| Profession/Occupation | ○ | ○ | ○ | ○ | ○ | ○ |
| Smoking | ○ | ○ | ○ | ○ | ○ | ○ |
| Exposure to environmental irritants | ○ | ○ | ○ | ○ | ○ | ○ |
| Medication history (ACE/ARBs) | ○ | ○ | ○ | ○ | ○ | ○ |
| Other | ○ | ○ | ○ | ○ | ○ | ○ |
| **Diagnostics/Testing:** |  |  |  |  |  |  |
| Blood tests | ○ | ○ | ○ | ○ | ○ | ○ |
| Physical examination | ○ | ○ | ○ | ○ | ○ | ○ |
| Pulmonary function studies | ○ | ○ | ○ | ○ | ○ | ○ |
| Chest radiography/Chest x-ray | ○ | ○ | ○ | ○ | ○ | ○ |
| Chest CT scans | ○ | ○ | ○ | ○ | ○ | ○ |
| Allergy assessments | ○ | ○ | ○ | ○ | ○ | ○ |
| Sinus CT scans | ○ | ○ | ○ | ○ | ○ | ○ |
| Spirometry, if asthma is suspected | ○ | ○ | ○ | ○ | ○ | ○ |
| Cardiac echo | ○ | ○ | ○ | ○ | ○ | ○ |
| **Referral/Treatment/ Management** |  |  |  |  |  |  |
| Referral to a specialist | ○ | ○ | ○ | ○ | ○ | ○ |
| Speech therapy | ○ | ○ | ○ | ○ | ○ | ○ |
| Trial of gabapentin | ○ | ○ | ○ | ○ | ○ | ○ |
| Trial of pregabalin | ○ | ○ | ○ | ○ | ○ | ○ |
| Trial of other medication (please specify): | ○ | ○ | ○ | ○ | ○ | ○ |

**21. To what extent do the approaches listed below describe or not describe the way you assess and treat chronic cough?**

|  |  |  |  |  |
| --- | --- | --- | --- | --- |
|  | Does not describe me at all (1) | Describes me slightly (2) | Describes me mostly (3) | Describes me completely (4) |
| I conduct my evaluation in phases, starting with medical history and few diagnostic tests with several trials of medications before I order more expensive tests or refer to a specialist | ○ | ○ | ○ | ○ |
| I refer all or most patients with chronic cough to a specialist for full evaluation | ○ | ○ | ○ | ○ |
| I follow the chronic cough treatment protocols we have at our practice (skip if not applicable) | ○ | ○ | ○ | ○ |
| I try to save money for the patient on upfront testing | ○ | ○ | ○ | ○ |
| I try to assess and treat the upper airway cough syndrome first and consider more expensive and invasive tests for those who are not improving | ○ | ○ | ○ | ○ |
| I assess where chronic cough is in level of importance in relation to other issues and needs of the patient | ○ | ○ | ○ | ○ |
| I try to assess for and to rule out most common causes of chronic cough through tests and trials of medications before I refer to a specialist | ○ | ○ | ○ | ○ |
| I follow evidence-based guidelines offered by the American College of Chest Physicians (ACCP) for management of chronic cough | ○ | ○ | ○ | ○ |
|  |  |  |  |  |

**22. To what extent would education in the following areas be beneficial to you?**

|  | Not at all beneficial (1) | Slightly beneficial (2) | Beneficial (3) | Mostly beneficial (4) | Very beneficial (5) |
| --- | --- | --- | --- | --- | --- |
| Evidence-based practices for management of chronic cough | ○ | ○ | ○ | ○ | ○ |
| Treatment options for chronic cough | ○ | ○ | ○ | ○ | ○ |
| Effects of chronic cough on patient's well-being | ○ | ○ | ○ | ○ | ○ |
| Recommended diagnostic tests | ○ | ○ | ○ | ○ | ○ |
| Patient education and communication strategies | ○ | ○ | ○ | ○ | ○ |
| Shared decision making | ○ | ○ | ○ | ○ | ○ |

**23. What factors do you consider when you evaluate (or assess) if your treatment for chronic cough is working for patients? (Select all that apply)**

- Patient's satisfaction with treatment (1)
- Symptom resolution (2)
- Adverse events (3)
- Effects of cough on important life domains such as physical functioning, sleep, mood, etc. (4)
- Patient's adherence to treatment (5)
- Economic factors such as treatment affordability or out of pocket expenses for the patient (6)
- Patient's disposition (7)
- Other (please specify): (8) ________________________________________________

**24. Who on your care team is typically involved in any aspects of care for patients with chronic cough? (Select all that apply)**

- Medical Assistants (1)
- Medical Students (2)
- Residents and/or Fellows (3)
- Nurses (4)
- Physician Assistants (5)
- Nurse Practitioners (6)
- Medical Laboratory staff (7)
- Pharmacists (8)
- Other primary care physicians (9)
- Other (please specify): (10) ________________________________________________

**25. If you refer your patients with chronic cough, how often do you refer your patients to the following specialists or services?**

|  | Never (1) | Rarely (2) | Sometimes (3) | Often (4) | Always(5) |
| --- | --- | --- | --- | --- | --- |
| Pulmonologist | ○ | ○ | ○ | ○ | ○ |
| Allergist | ○ | ○ | ○ | ○ | ○ |
| Gastroenterologist | ○ | ○ | ○ | ○ | ○ |
| Oncologist | ○ | ○ | ○ | ○ | ○ |
| Internal Medicine/ Infectious Disease | ○ | ○ | ○ | ○ | ○ |
| Otorhinolaryngology | ○ | ○ | ○ | ○ | ○ |
| Speech therapy | ○ | ○ | ○ | ○ | ○ |
| Behavioral therapy | ○ | ○ | ○ | ○ | ○ |
| Other (please specify): (9) | ○ | ○ | ○ | ○ | ○ |

**26. Thinking about referrals to specialists for your patients with chronic cough, to what extent are you satisfied with the following:**

|  | Not at all satisfied (1) | Slightly satisfied (2) | Satisfied (3) | Mostly satisfied (4) | Very satisfied (5) |
| --- | --- | --- | --- | --- | --- |
| The availability of specialists in your area | ○ | ○ | ○ | ○ | ○ |
| The access to specialists in your areas | ○ | ○ | ○ | ○ | ○ |
| Effectiveness of co-management process between you and a specialist for your patients with chronic cough | ○ | ○ | ○ | ○ | ○ |
| Communications and information sharing | ○ | ○ | ○ | ○ | ○ |
| Treatment recommendations provided by the specialist | ○ | ○ | ○ | ○ | ○ |

**27. How do you communicate with other specialists and services in the area? (Select all that apply)**

- Inter-EHR notes and communications (1)
- Referral letters (2)
- Phone call often initiated by me (3)
- Phone call often initiated by the specialist (4)
- Curb-side conversations (5)
- Post-visit letters (6)
- Document sent by fax (7)
- Other (please specify): (8) ________________________________________________
- None of the above (9)

**28. What are the barriers to providing comprehensive management of chronic cough?**

________________________________________________________________

________________________________________________________________

________________________________________________________________

**29. Please list your most pressing needs related to chronic cough evaluation, treatment, and management.**

________________________________________________________________

________________________________________________________________

________________________________________________________________

**30. Please provide a few words on why you chose to participate in this survey.**

________________________________________________________________

________________________________________________________________

________________________________________________________________

**31. If you would like to receive a $10 gift card for your participation, please provide the email address to which you would like the redemption information sent:**
